# Fyn-Mediated Paxillin Tyrosine 31 Phosphorylation Regulates Migration and Invasion of Breast Cancer Cells

**DOI:** 10.3390/ijms242115980

**Published:** 2023-11-05

**Authors:** Ying Zhang, Huanyu Zheng, Ming Xu, Noriko Maeda, Ryouichi Tsunedomi, Hiroko Kishi, Hiroaki Nagano, Sei Kobayashi

**Affiliations:** 1 Department of Molecular and Cellular Physiology, Graduate School of Medicine, Yamaguchi University, 1-1-1 Minami-Kogushi, Ube, Yamaguchi 755-8505, Japan; hirkishi@med.shimane-u.ac.jp; 2 Department of Gastroenterological, Breast and Endocrine Surgery, Graduate School of Medicine, Yamaguchi University, 1-1-1 Minami-Kogushi, Ube, Yamaguchi 755-8505, Japanhnagano@yamaguchi-u.ac.jp (H.N.)

**Keywords:** Fyn, paxillin Tyr31 phosphorylation, breast cancer, migration, invasion

## Abstract

Metastasis is the leading cause of death in breast cancer patients due to the lack of effective therapies. Elevated levels of paxillin expression have been observed in various cancer types, with tyrosine phosphorylation shown to play a critical role in driving cancer cell migration. However, the specific impact of the distinct tyrosine phosphorylation events of paxillin in the progression of breast cancer remains to be fully elucidated. Here, we found that paxillin overexpression in breast cancer tissue is associated with a patient’s poor prognosis. Paxillin knockdown inhibited the migration and invasion of breast cancer cells. Furthermore, the phosphorylation of paxillin tyrosine residue 31 (Tyr31) was significantly increased upon the TGF-β1-induced migration and invasion of breast cancer cells. Inhibiting Fyn activity or silencing Fyn decreases paxillin Tyr31 phosphorylation. The wild-type and constitutively active Fyn directly phosphorylate paxillin Tyr31 in an in vitro system, indicating that Fyn directly phosphorylates paxillin Tyr31. Additionally, the non-phosphorylatable mutant of paxillin at Tyr31 reduces actin stress fiber formation, migration, and invasion of breast cancer cells. Taken together, our results provide direct evidence that Fyn-mediated paxillin Tyr31 phosphorylation is required for breast cancer migration and invasion, suggesting that targeting paxillin Tyr31 phosphorylation could be a potential therapeutic strategy for mitigating breast cancer metastasis.

## 1. Introduction

Distant metastasis is the leading cause of death in breast cancer patients and so far, the mechanism of metastasis in breast cancer has not been fully clarified. Key steps in breast cancer metastasis are tumor cell migration and invasion, and these processes require cytoskeleton rearrangement [1,2,3]. Actin stress fibers are a higher-order cytoskeletal structure composed of cross-linked actin filament bundles which play a vital role in cell migration and invasion [4,5,6]. Actin stress fibers connect to focal adhesions and focal adhesion-associated proteins, such as paxillin and focal adhesion kinase, which are key regulators of actin cytoskeleton dynamics [7]. In addition, the RhoA/Rho-kinase signaling pathway is involved in actin stress fiber formation [8,9,10]. Fyn tyrosine kinase has also been demonstrated to be involved in actin stress fiber formation through the activation of Rho-kinase [11]. Recently, we identified paxillin as a novel signaling molecule that mediates SPC-induced actin stress fiber formation and cell migration by direct binding to the active Fyn [12]. 

Paxillin, one of the critical focal adhesion proteins, has multiple cell functions, such as migration and invasion [13,14], apoptosis [15,16], and autophagy [17,18]. Paxillin function is regulated by tyrosine phosphorylation and serine phosphorylation [19,20]. Activation of the corresponding tyrosine kinase or serine kinase by different stimuli and growth factors leads to the phosphorylation of paxillin and the initiation of related cellular functions. Cytoplasmic cyclin D1 together with its partner Cdk4 regulates cell invasion and metastasis through paxillin serine 83 phosphorylation [21]. Paxillin Tyr31 and Tyr118 phosphorylation are involved in EphB1-induced cell migration [22]; they also regulate HGF- or S1P-mediated lamellipodia formation [23] and LPS-mediated pulmonary vascular permeability and injury [24]. Recently, a study demonstrated the essential role of paxillin in promoting breast tumor collective cell invasion in breast cancer metastasis [25]. Although the multiple tyrosine phosphorylation of paxillin is important in controlling cell migration and invasion, the precise role of the single tyrosine phosphorylation of paxillin is unclear. Additionally, the underlying molecular mechanism by which paxillin tyrosine phosphorylation occurs has also not been completely clarified.

Fyn tyrosine kinase, a member of the Src family of protein tyrosine kinases (Src-TK), is overexpressed in breast cancer and promotes breast cancer cell proliferation, migration, and invasion [26,27]. Fyn promotes the mesenchymal phenotypes of basal-type breast cancer cells through the STAT5/NOTCH2 signaling node [27]. The tyrosine phosphorylation analysis of Src-TK revealed signaling network signatures in basal breast cancer cells and highlighted multiple kinases and their substrates as therapeutic targets and biomarkers [28]. Furthermore, a recent study suggested the involvement of the Fyn/paxillin signaling pathway in breast cancer cell migration [29]. While the roles of Fyn and paxillin in cancer cell migration have been reported, the exact mechanism through which Fyn influences paxillin function remains unclear. Specifically, the Fyn-mediated tyrosine phosphorylation of paxillin remains largely unexplored. 

In the present study, we investigated the role of paxillin Tyr31 phosphorylation in the migration and invasion of breast cancer cells. Furthermore, we found that paxillin Tyr31 phosphorylation is mediated by Fyn. We provide evidence that paxillin Tyr31 phosphorylation is involved in the migration and invasion of breast cancer cells, suggesting that paxillin Tyr31 phosphorylation could be a potential therapeutic target for mitigating breast cancer metastasis.

## 2. Results

### 2.1. High Paxillin Expression Is Correlated with Poor Prognosis of Breast Cancer Patients

**Figure 1:**
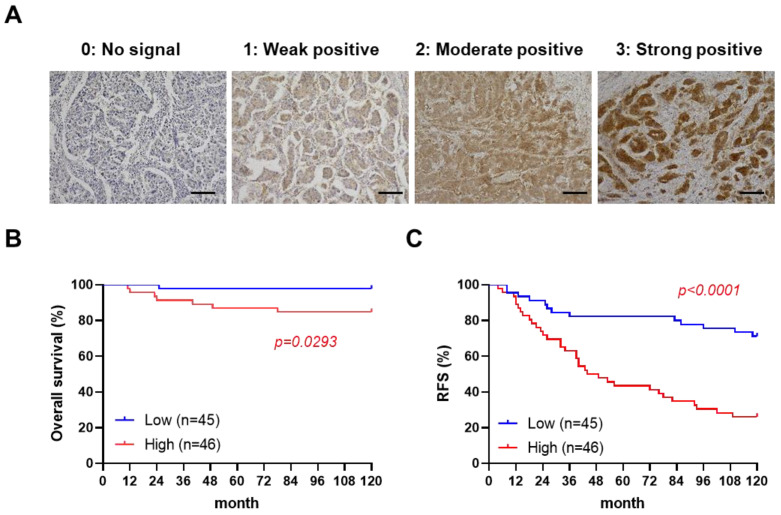
High paxillin expression is correlated with the poor prognosis of breast cancer patients. (**A**) Representative images of an immunohistochemistry analysis of paxillin in 91 breast cancer tissue samples (scale bars, 100 μm). (**B**,**C**) Kaplan–Meier overall survival curves (**B**) and recurrence-free survival (RFS) curves (**C**) for breast cancer patients in the paxillin-high group and paxillin-low group.

To examine the correlation between paxillin and a patient’s prognosis, we assessed the paxillin expression in clinical samples from 91 breast cancer patients (Supplementary Table S1). After confirmation by a professional pathologist, we assessed the paxillin expression levels in all breast cancer tissues and defined four levels as shown in Figure 1A. Subsequently, we divided patients into two groups according to their H-score: a paxillin-high group and a paxillin-low group, and compared the overall survival and recurrence-free survival of the two groups. Our results showed that a patient’s overall survival (Figure 1B) and recurrence-free survival (Figure 1C) in the paxillin-high group were significantly lower than those in the paxillin-low group. These results indicate that high paxillin expression is associated with a patient’s poor prognosis. 

### 2.2. Paxillin Is Involved in TGF-β1 Induced Migration and Invasion of Breast Cancer Cells 

**Figure 2:**
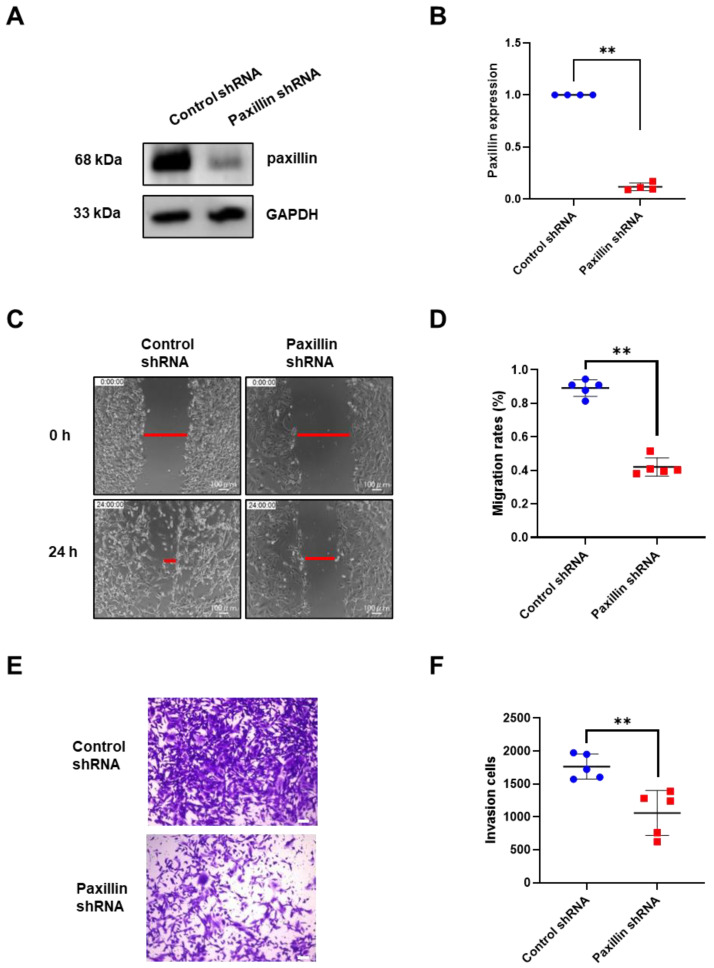
Paxillin knockdown inhibits the TGF-β1-induced migration and invasion of MDA-MB-231 cells. (**A**,**B**) Representative Western blot (**A**) and statistical analysis (**B**) showing the expression of paxillin in the control shRNA and paxillin shRNA transfected MDA-MB-231 cells. (**C**) Representative images showing the wound width in control shRNA and paxillin shRNA transfected MDA-MB-231 cells after stimulation with TGF-β1 for 24 h by wound healing assay, as indicated by the red lines. (**D**) Statistical analysis showing that paxillin knockdown inhibits the TGF-β1-induced migration of MDA-MB-231 cells. (**E**) Representative images showing the invasion of control shRNA and paxillin shRNA transfected MDA-MB-231 cells in the presence of TGF-β1 by transwell assay. Scale bar = 100 μm. (**F**) Statistical analysis showing that paxillin knockdown inhibits the TGF-β1-induced invasion of MDA-MB-231 cells. Data shown are the mean ± SEM. ** *p* < 0.01.

Migration and invasion are indicators of tumor malignancy [30]. To investigate the role of paxillin in the migration and invasion of breast cancer cells, we conducted a loss-of-function experiment. We first transfected paxillin shRNA particles to obtain the paxillin knockdown MDA-MB-231 cells (Figure 2A,B) and then assessed the TGF-β1-induced migration and invasion. As shown in Figure 2C,D, paxillin knockdown significantly inhibited TGF-β1-induced migration compared to control cells by a wound healing assay. Similarly, a transwell assay revealed that paxillin knockdown inhibited the TGF-β1-induced invasion of MDA-MB-231 cells (Figure 2E,F). These results suggest that paxillin plays a vital role in the TGF-β1-induced migration and invasion of breast cancer cells. 

### 2.3. Paxillin Tyr31 Phosphorylation Increases in TGF-β1-Induced Migration and Invasion

**Figure 3:**
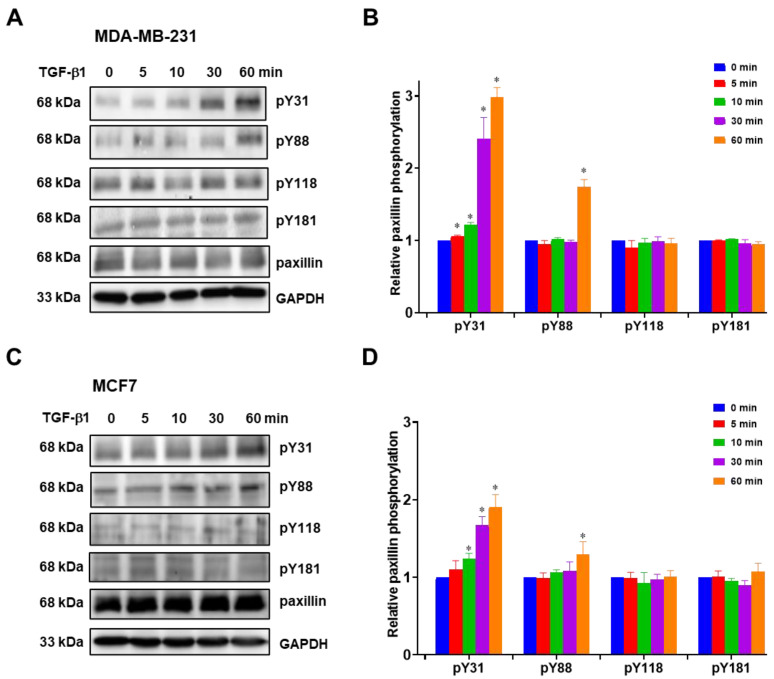
TGF-β1 stimulation increases paxillin Tyr31 phosphorylation. (**A**,**B**) Representative Western blot (**A**) and statistical analysis (**B**) showing the levels of Tyr31 phosphorylation (pY31), Tyr88 phosphorylation (pY88), Tyr118 phosphorylation (pY118), and Tyr181 phosphorylation (pY181) of paxillin after TGF-β1 stimulation in MDA-MB-231 cells. (**C**,**D**) Representative Western blot (**C**) and statistical analysis (**D**) showing the pY31, pY88, pY118, and pY181 levels of paxillin after TGF-β1 stimulation in MCF7 cells. Data shown are the mean ± SEM. *n* = 3, * *p* < 0.05 vs. corresponding 0 min in each group.

Previous studies have reported that paxillin tyrosine phosphorylation is linked to cancer metastasis [31,32,33]. Hence, we investigated paxillin tyrosine phosphorylation in the presence of TGF-β1 stimulation. However, multiple tyrosine sites on paxillin can be phosphorylated during cell migration [13,34]. In this study, we examined the tyrosine phosphorylation levels of paxillin at the Tyr31, Tyr88, Tyr118, and Tyr181 sites in the MDA-MB-231 cell line and MCF7 cell line. As shown in Figure 3A,B, TGF-β1 stimulation induced a time-dependent increase in paxillin Tyr31 phosphorylation in the MDA-MB-231 cell line. While paxillin Tyr88 phosphorylation slightly increased after TGF-β1 stimulation for 60 min, no changes were observed in paxillin Tyr118 and Tyr181 phosphorylation. Similar results were also observed in the MCF7 cell line (Figure 3C,D). These results suggest that paxillin Tyr31 and Tyr88 phosphorylation may play a crucial role in the TGF-β1-induced migration and invasion of breast cancer cells.

### 2.4. Fyn Directly Phosphorylates Paxillin Tyr31 

**Figure 4:**
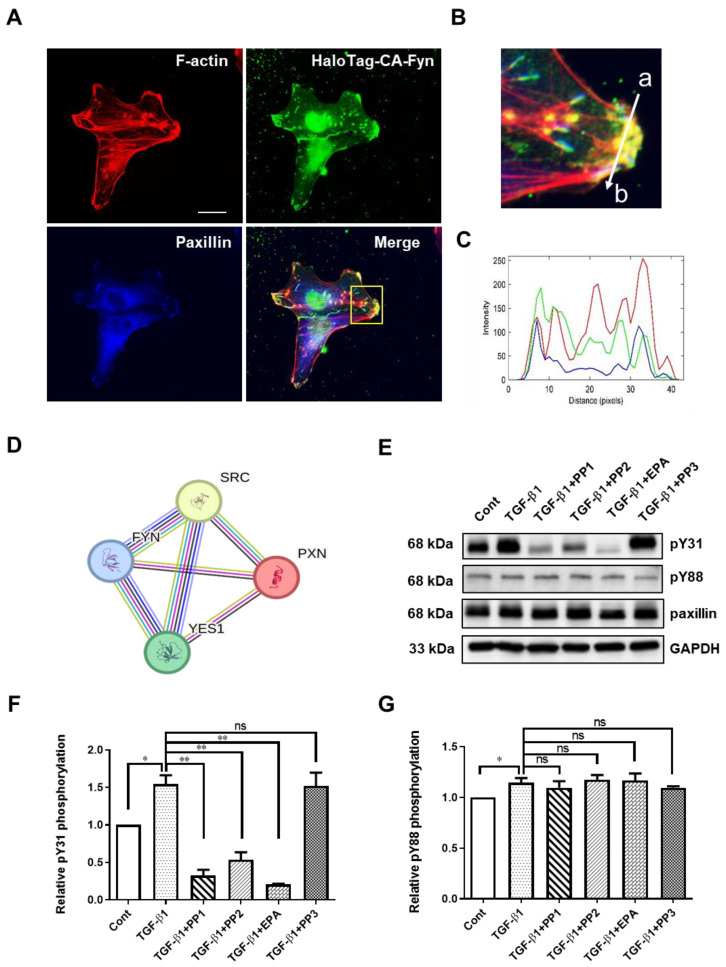
Exploration of Fyn-mediating TGF-β1-induced paxillin Tyr31 phosphorylation. (**A**) Immunofluorescent staining showing that the HaloTag constitutively active Fyn (HaloTag-CA-Fyn) and paxillin colocalize at the ends of actin stress fibers. Scale bar = 20 μm. (**B**) The enlarged image of the area outlined with a yellow line in (**A**). (**C**) Histogram showing the fluorescence intensity in each channel for HaloTag-CA-Fyn (green line), F-actin (red line), or paxillin (blue line) along the line (from a to b) indicated in (**B**). (**D**) The core network of the protein–protein interaction map of paxillin, Src, Fyn, and Yes. (**E**) Representative Western blot showing the effects of tyrosine kinase inhibitors on the TGF-β1-induced paxillin Tyr31 (pY31) and paxillin Tyr88 (pY88) phosphorylation in MDA-MB-231 cells. (**F**,**G**) Statistical analysis showing the effect of tyrosine kinase inhibitors on TGF-β1-induced pY31 (**F**) and pY88 (**G**) phosphorylation in MDA-MB-231 cells. Data shown are the mean ± SEM. *n* = 3, * *p* < 0.05; ** *p* < 0.01; ns, no significant.

To investigate the mechanism of paxillin Tyr31 and Tyr88 phosphorylation, we utilized the STRING database to analyze paxillin-interacting proteins. As shown in Figure 4D, the protein–protein interaction map of paxillin revealed its association with the Src family kinases, including Src, Fyn, and Yes. We recently reported that the direct interaction between active Fyn and paxillin regulates the migration of vascular smooth muscle cells [12]. This led us to ask whether Fyn could phosphorylate paxillin. Subsequent immunofluorescent results showed that the constitutively active Fyn colocalized with paxillin at the ends of actin stress fibers (Figure 4A–C), suggesting that Fyn may phosphorylate paxillin. To further explore this, we pretreated the cells with Src family kinase inhibitors, PP1 and PP2, and evaluated the TGF-β1-induced paxillin tyrosine phosphorylation. As shown in Figure 4E–G, PP1 and PP2 reduced TGF-β1-induced paxillin Tyr31 phosphorylation, but not Tyr88 phosphorylation. Additionally, the Fyn tyrosine kinase inhibitor EPA [35] strongly inhibited the TGF-β1-induced paxillin Tyr31 phosphorylation, but not Tyr88 phosphorylation. In contrast, PP3, the inactive control analog of PP1 and PP2, showed no effect on TGF-β1-induced paxillin Tyr31 phosphorylation or Tyr88 phosphorylation. These results suggest that Fyn is involved in TGF-β1-induced paxillin Tyr31 phosphorylation. 

**Figure 5:**
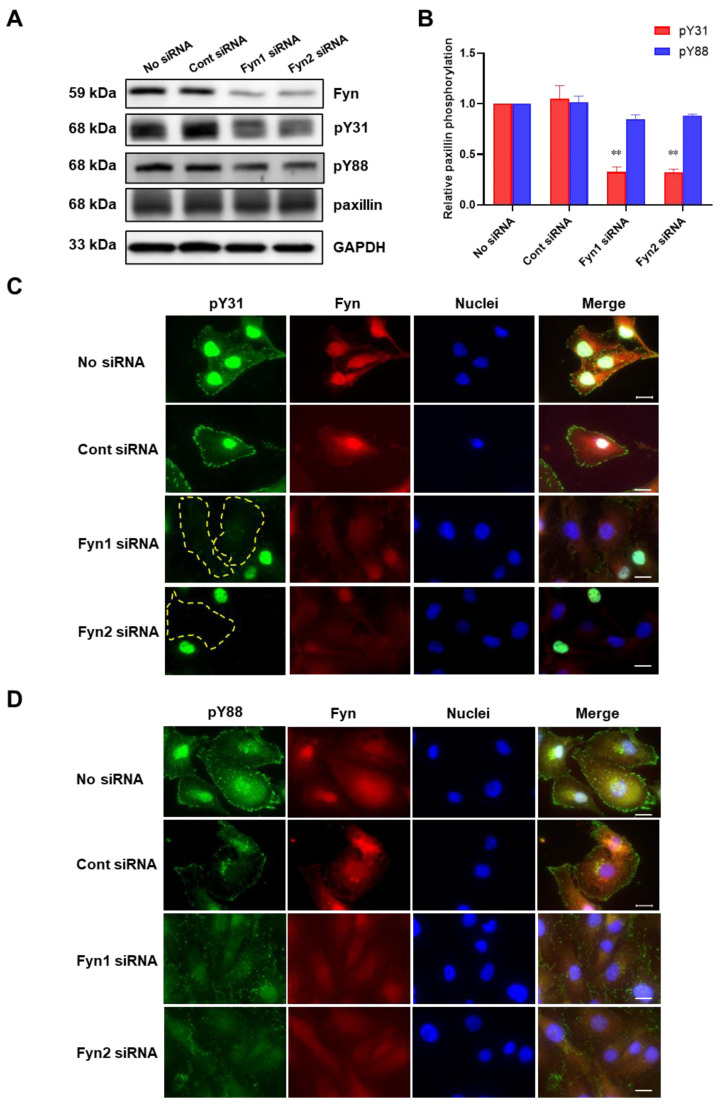
Fyn knockdown inhibits paxillin Tyr31 phosphorylation. (**A**,**B**) Representative Western blot (**A**) and statistical analysis (**B**) showing that Fyn knockdown inhibits TGF-β1-induced paxillin Tyr31 phosphorylation (pY31) but not paxillin Tyr88 phosphorylation (pY88) in MDA-MB-231 cells. Data shown are the mean ± SEM. *n* = 3, ** *p* < 0.01 vs. corresponding control siRNA group. (**C**,**D**) Immunofluorescent images showing that Fyn knockdown inhibits paxillin Tyr31 (**C**) but not paxillin Tyr88 (**D**) phosphorylation in MDA-MB-231 cells. The yellow dotted lines show that paxillin Tyr31 phosphorylation cannot be observed in cells. Scale bar = 20 μm.

**Figure 6:**
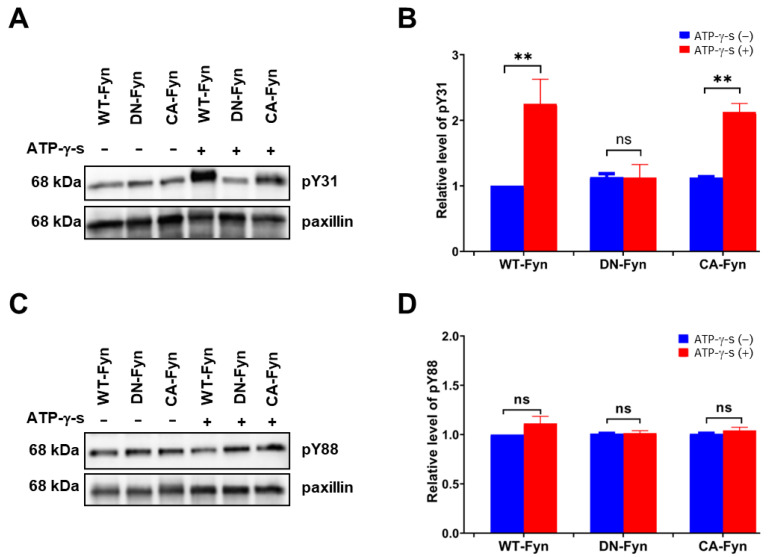
Fyn directly phosphorylates paxillin Tyr31 in vitro assay. Representative Western blot and statistical analysis showing that wild-type Fyn (WT-Fyn) and constitutively active Fyn (CA-Fyn) directly phosphorylate the Tyr31 (**A**,**B**) but not Tyr88 (**C**,**D**) of paxillin in the presence of ATP-γ-s. Data shown are the mean ± SEM. *n* = 3, ** *p* < 0.01; ns: no significant. DN-Fyn, dominant negative Fyn.

To further confirm whether Fyn phosphorylates paxillin Tyr31 or not, we examined the effect of Fyn knockdown on paxillin tyrosine phosphorylation. Our results showed that Fyn knockdown inhibited paxillin Tyr31 phosphorylation, but not Tyr88 phosphorylation (Figure 5A,B). Additionally, immunofluorescent staining also revealed that paxillin Tyr31 phosphorylation was almost completely inhibited in Fyn siRNA transfected cells (Figure 5C) while paxillin Tyr88 phosphorylation remained unchanged (Figure 5D). These results indicate that Fyn phosphorylates paxillin Tyr31. In addition, as shown in Figure 6A,B, wild-type Fyn (WT-Fyn) and constitutively active Fyn (CA-Fyn), but not dominant negative Fyn (DN-Fyn), significantly increased paxillin Tyr31 phosphorylation in the presence of ATP in vitro. However, no significant changes in paxillin Tyr88 phosphorylation were observed (Figure 6C,D). These results indicate that Fyn directly phosphorylates paxillin Tyr31 but not Tyr88.

### 2.5. Non-Phosphorylatable Paxillin Tyr31 Mutant Attenuates TGF-β1-Induced Migration and Invasion

**Figure 7:**
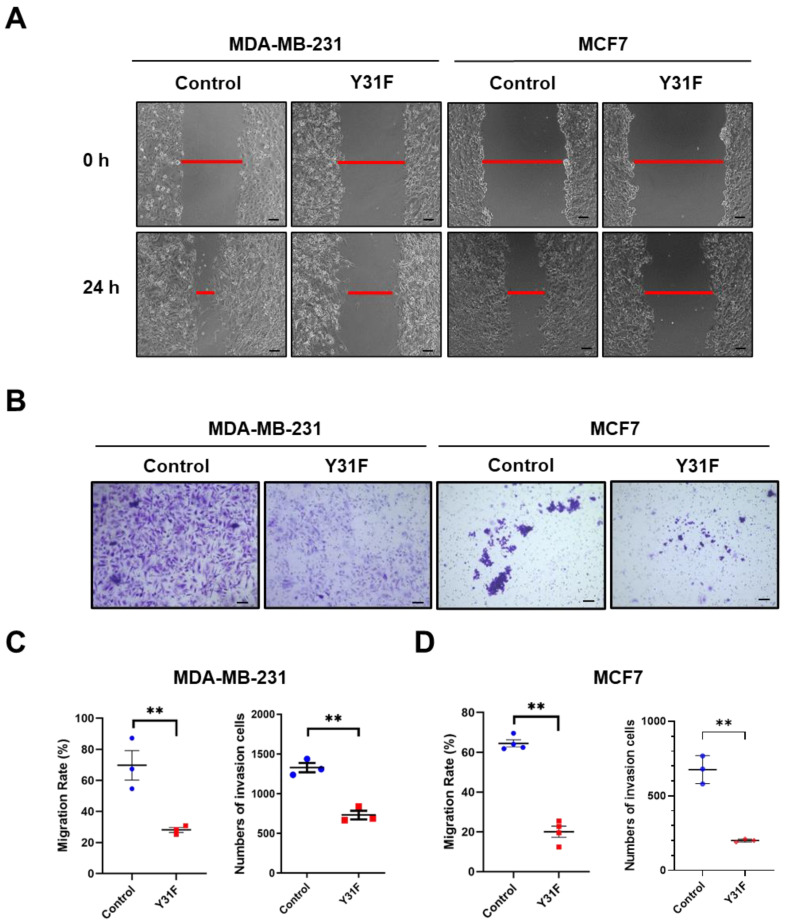
Paxillin Y31F overexpression attenuates TGF-β1-induced migration and invasion. (**A**) Representative images showing wound width by wound healing assay in paxillin Y31F-transfected MDA-MB-231 cells and MCF7 cells at 24 h, as indicated by the red lines. Scale bar = 100 μm. (**B**) Representative images showing invasion cells in paxillin Y31F-transfected MDA-MB-231 cells and MCF7 cells by transwell assay. Scale bar = 100 μm. (**C**) Statistical analysis showing the effects of paxillin Y31F on the TGF-β1-induced migration and invasion of MDA-MB-231 cells. Data shown are the mean ± SEM. ** *p* < 0.01. (**D**) Statistical analysis showing the effects of paxillin Y31F on TGF-β1-induced migration and invasion of MCF7 cells. Data shown are the mean ± SEM. *n* = 3 or 4, ** *p* < 0.01.

To clarify the role of paxillin Tyr31 phosphorylation in the migration and invasion of breast cancer cells, we generated a non-phosphorylatable paxillin Tyr31 mutant by replacing the tyrosine residue with phenylalanine (Y31F). We then examined the effect of paxillin Y31F overexpression on the migration and invasion of breast cancer cells. Our results showed that paxillin Y31F overexpression inhibited the TGF-β1-induced migration (Figure 7A,C,D) and invasion (Figure 7B–D) of both MDA-MB-231 and MCF7 cells, suggesting that paxillin Tyr31 phosphorylation plays an important role in the migration and invasion of breast cancer cells.

### 2.6. Non-Phosphorylatable Paxillin Tyr31 Mutant Attenuates TGF-β1-Induced Actin Stress Fiber Formation

**Figure 8:**
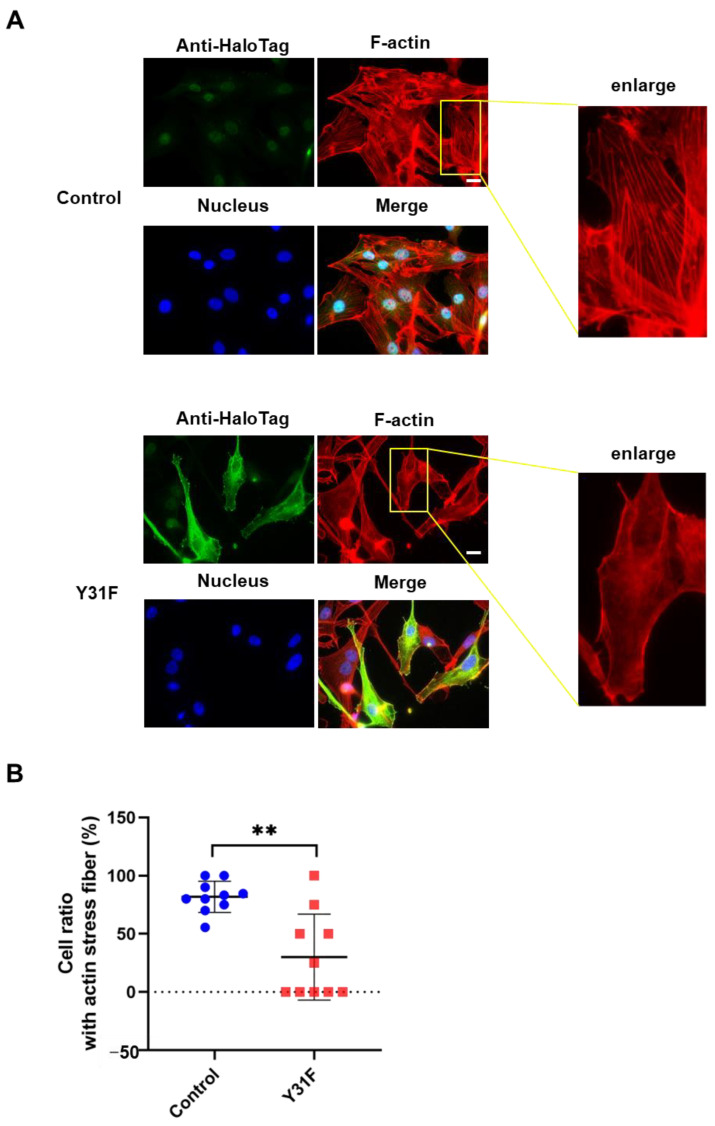
Paxillin Y31F overexpression inhibits TGF-β1-induced actin stress fiber formation. (**A**) Representative images showing the TGF-β1-induced actin stress fiber formation in paxillin Y31F-transfected MDA-MB-231 cells and control cells. Scale bar = 20 μm. (**B**) Statistical analysis showing the effect of paxillin Y31F on the TGF-β1-induced actin stress fiber formation in MDA-MB-231 cells. Ten fields of view from three independent experiments were randomly selected and then the ratio of the cells with actin stress fiber formation in each field was calculated. At least 30 cells were counted. Data shown are the mean ± SEM. ** *p* < 0.01.

Actin cytoskeleton remodeling has a fundamental role in cancer cell dynamics, such as migration and invasion [2,36]. To investigate whether paxillin Tyr31 phosphorylation impacts actin cytoskeleton remodeling, we observed the actin stress fiber formation in MDA-MB-231 cells overexpressed with paxillin Y31F. As shown in Figure 8A,B, TGF-β1 induced a remarkable actin stress fiber formation in control cells. However, paxillin Y31F overexpression inhibited TGF-β1-induced actin stress fiber formation. These findings demonstrate that paxillin Tyr31 phosphorylation is involved in actin stress fiber formation, leading to the subsequent migration and invasion of breast cancer cells.

## 3. Discussion

In the present study, we sought to understand the mechanism by which paxillin is involved in the migration and invasion of breast cancer cells and to determine the critical role of paxillin Tyr31 phosphorylation on the migration and invasion of breast cancer cells. We provide direct evidence that Fyn tyrosine kinase phosphorylates paxillin Tyr31. Our findings suggest that paxillin Tyr31 phosphorylation mediated by Fyn plays an important role in the migration and invasion of breast cancer cells by participating in the formation of actin stress fibers. Paxillin Tyr31 phosphorylation could be a potential target site for the treatment of breast cancer metastasis.

**Figure 9:**
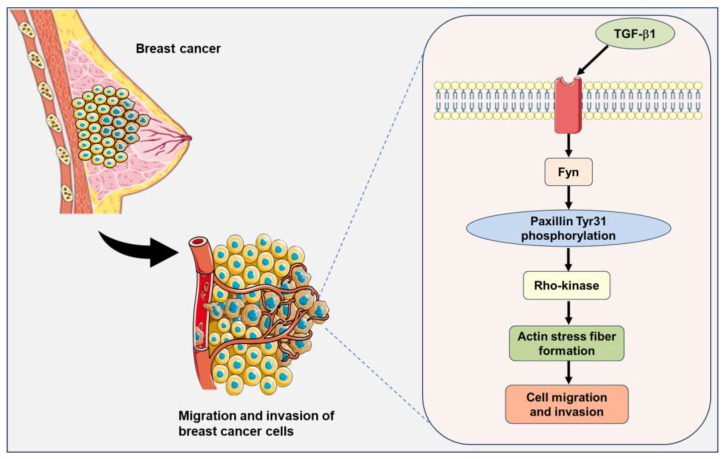
A signaling pathway showing that Fyn-mediated paxillin Tyr31 phosphorylation participates in the TGF-β1-induced migration and invasion of breast cancer cells. The illustrations including receptors, breast cancer cells, and plasma membranes are obtained from Smart Servier Medical Art, provided by Servier, licensed under a Creative Commons Attribution 3.0 unported license.

Paxillin has been shown to be highly expressed in many malignant tumors and is frequently associated with progression and poor patient outcomes, as seen in gastric cancer [37], lung cancer [38,39], esophageal cancer [40], and colorectal cancer [41]. Src family tyrosine kinases phosphorylate paxillin and a broad-spectrum Src family tyrosine kinase inhibitor is currently used in the clinic to treat cancer [42]. However, paxillin has many tyrosine phosphorylation sites and which site is phosphorylated by the special kinase has not been completely identified. Iwasaki T et al. reported that lysophosphatidic acid (LPA) stimulation induced the paxillin Tyr31 phosphorylation involved in the migration of rat ascites hepatoma MM1 cells [43]. Fu P et al. found that LPS-induced paxillin Tyr31 phosphorylation is mediated by c-Abl tyrosine kinase but not by Src and focal adhesion kinase [24]. In our recent study, we found that active Fyn binds directly to paxillin, and this direct Fyn-paxillin binding further activates Rho-kinase, ultimately leading to the formation of actin stress fibers that contribute to cell migration [12]. Here, we further elucidated the physiological significance of Fyn binding to paxillin, which is to directly phosphorylate paxillin Tyr31, thereby regulating actin stress fiber formation and participating in the migration and invasion of breast cancer cells. Our findings demonstrate that the overexpression of paxillin Y31F inhibits actin stress fiber formation, indicating that Fyn-mediated paxillin Tyr31 phosphorylation plays a crucial role in actin stress fiber formation through the activation of Rho-kinase (Figure 9). Consistently with our results, Azuma K et al. suggested that the enhanced activity of Src family tyrosine kinases and paxillin overexpression synergistically contribute to the high metastatic potential of human osteosarcoma through the hyperphosphorylation of paxillin [32]. These findings, together with our present data and previous reports, show the importance of paxillin and paxillin Tyr31 phosphorylation in breast cancer metastasis.

TGF-β1, a cytokine, regulates diverse cellular biological processes. TGF-β1 has been reported to act as a tumor suppressor in early-stage tumors but paradoxically functions as a potent tumor promoter in advanced cancers [44]. Many studies have demonstrated that TGF-β1 induces the migration and invasion of breast cancer cells [45,46,47,48]. However, the precise mechanism underlying the TGF-β1-induced migration and invasion in breast cancer cells has not been fully elucidated. Fyn tyrosine kinase has been reported to be involved in many TGF-β1-induced cellular biological processes. TGF-β1 rapidly induces Fyn activation and subsequently induces mitochondrial ROS (mtROS) production and genetic damage in human bronchial epithelial cells [49]. Fyn kinase plays an important role in the modulation of actin cytoskeletal dynamics and the TGF-β-induced migration of mast cells [50]. Additionally, paxillin has been demonstrated to be involved in actin stress fiber formation and the migration of vascular smooth muscle cells as an upstreaming molecule of Rho-kinase [12]. Based on these reports, we speculate that paxillin Tyr31 phosphorylation is involved in the actin stress fiber formation through the TGF-β1/Fyn/paxillin/Rho-kinase signaling pathway, thereby leading to the migration and invasion of breast cancer cells (Figure 9).

Previous studies have revealed the important role of the simultaneous phosphorylation of multiple tyrosine sites of paxillin in facilitating tumor cell migration and invasion. Specifically, paxillin Tyr31 and Tyr118 phosphorylation induced by activated integrins have been shown to promote the migration of cancer cells [43,51,52]. Additionally, a recent study demonstrated that hesperetin inhibits the migration and invasion of breast cancer cells by suppressing the phosphorylation of paxillin at Tyr31, Tyr88, and Tyr118 [29]. Despite these insights linking paxillin tyrosine phosphorylation to cell migration, the relative importance of each phosphorylation site remains unclear. To the best of our knowledge, it is unclear whether individual tyrosine residues hold distinct functional roles when phosphorylated. Our recent research established that the direct interaction between Fyn and paxillin is crucial for cell migration [12,53]. However, the precise details of how this interaction leads to the tyrosine phosphorylation of paxillin, and specifically which site is phosphorylated, remain unresolved. In this study, we provide the initial evidence that Fyn directly mediates paxillin Tyr31 phosphorylation, promoting breast cancer cell migration. The expression of non-phosphorylatable paxillin Y31F leads to the inhibition of actin stress fiber formation, as well as impairs cell migration and invasion. These findings indicate the significance of the Tyr31 phosphorylation of paxillin, establishing it as a pivotal event in the regulation of cell migration.

In summary, our study provides important new insights into the role of Fyn in governing paxillin activity during breast cancer cell migration. The phosphorylation of paxillin at Tyr31 by Fyn plays a pivotal role in driving the migration and invasion of breast cancer cells. These findings provide a new theoretical foundation for the treatment of breast cancer metastasis.

## 4. Materials and Methods

### 4.1. Reagents and Antibodies

The following reagents were used in this study. Rhodamine phalloidin (Life technologies, Tokyo, Japan), Src family tyrosine kinase inhibitors PP1 and PP2, and the inactive analog PP3 were purchased from Sigma; EPA was purchased from Santa Cruz Biotechnology. Transforming growth factor-β1 (TGF-β1) was purchased from Wako (209-16544, Osaka, Japan). It was made as 40-μg/mL stock and stored at −80 °C before use. A final concentration of 20 ng/mL of TGF-β1 was used in the present study. All other reagents were purchased from Sigma (Sigma-Aldrich, St. Louis, MO, USA).

The following antibodies were used in this study. Primary antibodies including anti-Fyn (BD Biosciences, San Jose, CA, USA), anti-paxillin (BD Biosciences, San Jose, CA, USA or Abcam, Cambridge, UK), anti-pY31-paxillin (Abcam, Cambridge, UK), anti-pY88-paxillin (Bioss, Woburn, MA, USA), anti-pY118-paxillin (BD Biosciences, San Jose, CA, USA), anti-pY181-paxillin (Abcam, Cambridge, UK), anti-glyceraldehyde 3 phosphate dehydrogenase (GAPDH) (FujiFilm, Tokyo, Japan), anti-HaloTag (Promega, Madison, WI, USA), and secondary antibodies against anti-mouse HRP-conjugated IgG (Promega, Madison, WI, USA) and anti-rabbit HRP-conjugated IgG (Promega, Madison, WI, USA) were used.

### 4.2. Human Breast Cancer Tissue Samples 

Paraffin-embedded breast cancer tissues from 91 female patients were included in this study. All patients received cancer therapy at the Yamaguchi University Hospital (Ube, Yamaguchi, Japan) between September 1995 and December 2019. The study protocols were approved by the Institutional Review Board of Yamaguchi University/Graduate School of Medicine (H2022-177), and the study was conducted in accordance with the Declaration of Helsinki. Each patient provided written informed consent to participate in this study.

### 4.3. Immunohistochemical (IHC) Staining and Data Analysis 

Paxillin expression in breast cancer tissues was detected by immunohistochemical (IHC) staining as described below. The staining was performed on 4 μm formalin-fixed paraffin-embedded slides. The samples were deparaffinized in xylene and hydrated in graded alcohols; antigen retrieval was performed in EDTA buffer (pH 9.0) and heated in a microwave at 95 °C for 40 min. After endogenous peroxidase and nonspecific protein blocking, the slides were applied with monoclonal anti-paxillin antibody (1:600, Abcam, ab32115) and incubated at 4 °C overnight. The next day, after washing with PBS three times, the slides were applied with the corresponding secondary antibody for 30 min. Finally, the samples were visualized with 3,3′-diaminobenzidine chromogen and counterstained with Mayer’s hematoxylin. 

The histochemical scoring (H-score) assessment method was used to assess the paxillin expression level. In brief, the method focused on both the staining intensity and the percentage (P) of stained cells at each intensity level. The intensity values are indicated as 0 (no evidence of staining), 1 (weak staining), 2 (moderate staining), and 3 (strong staining). The percentage values vary from 0% to 100%. The final H-score = 0 × P_0_ + 1 × P_1_ + 2 × P_2_ + 3 × P_3_ (range from 1 to 300). Immunohistochemical staining was visualized by KEYENCE microscope (KEYENCE, Osaka, Japan) and confirmed by a professional pathologist. To verify the association between survival or the recurrence-free survival rate and paxillin expression, patients were divided into two groups (paxillin-low and paxillin-high) based on their H-score. The median value of paxillin expression data was used as the cutoff value (H-score = 145).

### 4.4. Cell Culture and Paxillin shRNA Transfection

Cells from the human breast cancer cell line MDA-MB-231 from DS Pharma Biomedical (Osaka, Japan) were cultured in L-15 medium supplemented with 15% FBS, 2 mM glutamine, 50 U/mL penicillin, and 50 μg/mL streptomycin at 37 °C in a humidified atmosphere of 5% CO_2_ and 95% air. Cells from the human breast cancer cell line MCF7 were provided from Yamaguchi University Center for Gene Research and cultured at 37 °C in Dulbecco’s Modified Eagle’s Medium (DMEM, Thermo Fisher Scientific, Waltham, MA, USA) supplemented with 10% FBS, 50 U/mL penicillin, and 50 μg/mL streptomycin.

The lentivirus containing paxillin short hairpin RNA (shRNA) or non-target oligonucleotides was obtained from Santa Cruz (Dallas, CA, USA). Paxillin shRNA lentiviral particles (sc-29439-V, Santa Cruz, Dallas, CA, USA) or control shRNA lentiviral particles (sc-108080, Santa Cruz, Dallas, CA, USA) were transfected into MDA-MB-231 cells according to the manufacturer’s instructions as described in our previous report [12]. 

Fyn1 and Fyn2 siRNAs were synthesized as previously described [9,11]. Control (non-silencing) siRNA was obtained from Qiagen. Cells were transfected with 40 nM siRNA using a siRNA transfection reagent (sc-29528, Santa Cruz, Dallas, CA, USA) according to the manufacturer’s instructions. After the siRNA transfection, stimulation with TGF-β1 and the determination of knockdown efficiency by Western blot analysis followed.

### 4.5. Plasmid Constructs and Transfection

Human cDNA encoding constitutively active Fyn (CA-Fyn, Y530F) subcloned into the pFC14AHaloTag vector (Promega, Madison, WI, USA) was used for transfection [12]. The HaloTag full-length paxillin (FHC10852, 1–1671 bp) plasmid was purchased from Promega. A non-phosphorylated mutant of HaloTag paxillin Y31F was obtained by Promega mutating tyrosine to phenylalanine at Tyr31 of the HaloTag full-length paxillin.

For HaloTag paxillin Y31F transfection, the Lipofectamine™ LTX Reagent with PLUS™ Reagent (Thermo Fisher Scientific, Waltham, MA, USA) was used according to the manufacturer’s protocol. Briefly, 3 × 10^5^ cells were seeded into a six-well plate in a medium without penicillin and streptomycin. After 24 h, MDA-MB-231 cells or MCF7 cells were transfected with Lipofectamine™ LTX Reagent (6 μL) and the paxillin Y31F plasmid (2 μg) in the presence of PLUS™ Reagent (2 μL). The cell transfection efficiency was analyzed with HaloTag TMR Direct Ligand (Promega, Madison, WI, USA) after 24 h transfection. 

### 4.6. Protein Expression and Purification

The baculovirus expression system was used to express recombinant Fyn protein as previously described [12]. In brief, after Sf9 cells (Invitrogen, Carlsbad, CA, USA) were infected with viruses carrying the recombinant Fyn for 72 h, the cells were pelleted by centrifugation and suspended in a buffer containing 25 mM Tris-HCl (pH 7.4), 10% glycerol, 150 mM NaCl, 40 mM imidazole (pH 7.4), 1% NP-40, 10 μg/mL aprotinin, 5 μg/mL leupeptin, 7 mM β-mercaptoethanol, and His-Tag protein protease inhibitor cocktail (Sigma-Aldrich, St. Louis, MA, USA). After the suspension was sonicated and centrifuged, the supernatant was filtered with a 0.22 μm filter (Millipore, Burlington, MA, USA) and then applied to the His-Trap HP affinity column chromatography (Qiagen, Hilden, Germany) to separate and purify the recombinant Fyn. 

HaloTag fusion full-length paxillin (1–1671 bp) was cloned into the pFN18AHaloTag T7 Flexi vector (Promega, Madison, WI, USA) and expressed and purified as described in our previous report [12]. Briefly, after the lysis of harvested Single Step (KRX) Competent Cells by sonication in the HaloTag purification buffer (50 mM HEPES pH 7.5, 150 mM NaCl, 1 mM DTT, and 0.05% IGEPAL-CA-630), ATP (final concentration: 2 mM) and MgCl_2_ (final concentration: 10 mM) were added to remove the chaperones bound to the HaloTag fusion protein. Lysates were then centrifuged at 10,000× *g* for 20 min. The supernatant was then mixed with HaloLink^TM^ resin (Promega, Madison, WI, USA) for 1 h at 4 °C on a rotating wheel. HaloTag was removed by adding a TEV protease lysis solution (Promega, Madison, WI, USA) to the settled resin. Finally, the HisLink^TM^ resin was used to remove TEV protease, and paxillin was obtained after centrifugation. 

### 4.7. In Vitro Paxillin TYROSINE Phosphorylation by Fyn

Wild-type Fyn (WT-Fyn), CA-Fyn, or dominant negative Fyn (DN-Fyn) (10 ng) was incubated with recombinant paxillin (1 μg) in a kinase buffer (50 mM HEPES {pH 7.4}, 15 mM MgCl_2_, and 200 μM sodium vanadate) containing 100 μM ATP-γ-s (ab138911, Abcam, Cambridge, UK) at 30 °C for 30 min. Phosphorylated proteins were separated by SDS-PAGE and analyzed by Western blot with the corresponding antibody. A control experiment was performed under the same conditions in which ATP-γ-s was omitted. 

### 4.8. Western Blotting

Cell samples were lysed in lysis buffer (50 mM Tris-HCl, pH 7.4; 150 mM NaCl; 0.5% NP-40; 0.1% Triton X-100; 1 mM DTT; 0.2 mM Na_3_VO_4_; 10 mM NaF; 1 mg/mL aprotinin; and protease inhibitor cocktail (Sigma)) and then kept at 95 °C for 5 min for Western blot analysis. Cell samples were separated by 10% SDS-PAGE, then proteins were transferred to a PVDF membrane (Hybond-P, GE Healthcare Bioscience, Piscataway, NJ, USA) and to carry out the Western blot [12,54] with the appropriate primary antibodies and appropriate secondary antibodies. All antibodies were diluted with Tris-buffered saline with 0.05% Tween-20 (TBS-T). GAPDH was used as a loading control. The protein bands were visualized with the Super Signal West Pico chemiluminescence substrates (Thermo Fisher Scientific, Waltham, MA, USA) and evaluated using Quantity One 4.6.2 with ChemiDoc XRS-J software (Bio-Rad, Hercules, CA, USA).

### 4.9. Immunofluorescent Staining

Immunofluorescence staining was carried out according to our previous report [12,54]. Briefly, cells were fixed in 4% paraformaldehyde for 10 min, permeabilized with 0.1% Triton X-100 for 2 min, and blocked with a Blocking One Histo Solution (Nacalai, Kyoto, Japan) for 60 min. After treatment with primary antibodies or rhodamine-conjugated phalloidin (1:100, Thermo Fisher Scientific, Waltham, MA, USA) for staining F-actin and fluorescently labeled secondary antibodies (anti-rabbit or anti-mouse Alexa Fluor 488, 1:100, Thermo Fisher Scientific, Waltham, MA, USA), cells were mounted with PermaFluor aqueous mounting medium (Thermo Fisher Scientific, Waltham, MA, USA). Cells were observed using a fluorescence microscope (KEYENCE Biorevo BZ-9000, Osaka, Japan). Actin stress fiber formation was observed and quantified as described previously [12].

### 4.10. Wound-Healing Assay 

A wound-healing assay was performed as described previously [55]. MDA-MB-231 cells or MCF7 cells were grown in 35-mm glass-based dishes (Iwaki, Tokyo, Japan). When cell confluence reached 90–100%, the cells were starved with FBS-free medium for 24 h. Then, the cells were wounded using 200 μL micropipette tips and then TGF-β1 (20 ng/mL) was added. Cell migration was recorded under a fluorescence microscope with a phase contrast model (KEYENCE, Osaka, Japan). The data were used for quantitative analyses of cell migration using NIH Image J software 1.53t. The migration rate was calculated as follows: The migration rate (%) = (scratch width at 0 h − scratch width at 24 h)/scratch width at 0 h × 100%. 

### 4.11. Transwell Invasion Assay

The invasion of MDA-MB-231 cells or MCF7 cells was determined using a corning invasion chamber 24-well plate (354480, Corning BioCoat, Manassas, VA, USA) as described previously [51]. A total of 1 × 10^6^ cells were placed into the upper chamber coated with an extracellular matrigel matrix (BD Biosciences, San Jose, CA, USA). The medium with TGF-β1 (20 ng/mL) added was filled in the lower compartment. After culturing for 24 h (for MDA-MB-231 cells) or 48 h (for MCF7 cells), the invading cells on the underside of the membrane were fixed with formaldehyde (4%) for 10 min and stained with crystal violet (0.1%) for 30 min. Cells were visualized under a bright-field microscope at a magnification of 10× (KEYENCE, Osaka, Japan) and counted by NIH Image J software 1.53t.

### 4.12. Statistical Analysis

All statistical analyses were carried out using GraphPad Prism 8.0.2. The data shown represent the mean ± SEM. For comparisons between the two groups, the significance of differences was determined using a Student’s *t*-test. For comparisons of multiple groups, an ANOVA assay was used to determine the significance of differences. *p* < 0.05 was regarded as statistically significant.

## 5. Conclusions

Our study showed that paxillin Tyr31 phosphorylation plays an important role in the migration and invasion of breast cancer cells. Thus, targeting paxillin Tyr31 phosphorylation could be a promising therapeutic strategy for the treatment of breast cancer metastasis. Future studies will focus on understanding how paxillin tyrosine phosphorylation is able to coordinate and integrate the activity of Rho-kinase to optimize actin stress fiber formation.

## Data Availability

The data presented in this study are available within the article text and figures.

## Supplementary Materials

The following supporting information can be downloaded at: https://www.mdpi.com/article/10.3390/ijms242115980/s1.
