# Supplementary material for: Fyn-Mediated Paxillin Tyrosine 31 Phosphorylation Regulates Migration and Invasion of Breast Cancer Cells
Source: Int J Mol Sci. 2023 Nov 5;24(21):15980. doi: 10.3390/ijms242115980 (PMC10647795; doi:10.3390/ijms242115980)
Supplement: Supplementary file 1 [file ijms-24-15980-s001.zip › ijms-2679821-supplementary.pdf]

Supplementary Table S1

The expression level of paxillin and clinicopathological parameters in breast cancer patients (n = 91). Patients were divided into two groups (high group and low group) based on H-score. The median value of paxillin expression data was used as the cutoff value (H-score = 145).

Clinicopathological factors	Paxillin expression level	
	High (46) (H-score > 145)	Low (45) (H-score < 145)
Age	58.06±11.75	53.84±11.99
Stage		
0	2	0
I	19	24
II	25	21
III	0	0
IV	0	0
T stage		
Tis	2	0
T1	22	26
T2	18	17
T3	2	2
T4	0	0
N stage		
N0	36	40
N1	9	5
N2	1	0
N3	0	0
